# Evaluation of a mobile health intervention to support asthma self-management and adherence in the pharmacy

**DOI:** 10.1007/s11096-019-00798-3

**Published:** 2019-04-27

**Authors:** Richelle C. Kosse, Marcel L. Bouvy, Tjalling W. de Vries, Ellen S. Koster

**Affiliations:** 1 Division of Pharmacoepidemiology and Clinical Pharmacology, Utrecht Institute for Pharmaceutical Sciences (UIPS), Faculty of Science, Utrecht University, PO Box 80082, 3508 TB Utrecht, The Netherlands; 2 Department of Paediatrics, Medical Centre Leeuwarden (MCL), Leeuwarden, The Netherlands

**Keywords:** Adherence, Adolescents, Evaluation, mHealth, Netherlands, Pharmacists

## Abstract

*Background* Several effective mobile health (mHealth) interventions have been developed to support patients with their medication use, however hardly any is implemented in clinical practice. Process evaluations and user experiences are therefore important for further implementation. *Objective* To explore experiences, barriers, and facilitators of pharmacists and patients towards the use of the interactive ADolescent Adherence Patient Tool (ADAPT). In addition, the perceptions of pharmacists towards mHealth interventions in general were explored. *Setting* Dutch community pharmacies. *Methods* Pharmacists (N = 24) and adolescent asthma patients (N = 87; age 12–18) completed a questionnaire about the ADAPT intervention. Pharmacists who did not have access to the ADAPT intervention (N = 26) completed a questionnaire on their perceptions towards mHealth. *Main outcome measure* Experiences, barriers, and facilitators of pharmacists and patients. *Results* Most patients (78%) would recommend the ADAPT intervention to others, and thought that the pharmacy was the right place for mHealth aiming to support adherence (63%). The possibility to monitor asthma symptoms was highly appreciated by patients and pharmacists. Pharmacists were satisfied with ADAPT intervention (96%), and using the intervention was not time consuming (91%). The ADAPT intervention promoted contact with patients (74%) and facilitated the healthcare providing role of pharmacists (83%). Pharmacists who did not have access to the ADAPT intervention mentioned time constraints and funding as main barriers for using mHealth. *Conclusion* Pharmacists and patients perceived many beneficial effects and were positive about the the use of the interactive ADolescent Adherence Patient Tool (ADAPT) intervention. This study emphasizes opportunities for mHealth in improving the quality of care, which supports the need for further implementation in clinical practice.

**Electronic supplementary material:**

The online version of this article (10.1007/s11096-019-00798-3) contains supplementary material, which is available to authorized users.

## Impacts on practice

- A mHealth interventions supporting self-management and adherence can be used in daily pharmacy practice.
- An interactive mHealth intervention supports contact between pharmacists and patients and has thereby the potential to improve pharmaceutical care.
- Proper reimbursement of mHealth will support further implementation and integration of mHealth in the pharmacy.

## Introduction

Suboptimal adherence is a major problem among patients with chronic conditions, negatively affecting health outcomes and treatment costs. On average, 50% of patients fail to adhere to the recommendations of their healthcare provider [1, 2]. Information and communication technologies (ICT) are increasingly used to support patients with chronic conditions [3–5], in particular the use of mobile health (mHealth) have increased. Mobile device technologies, such as smartphone applications (apps), may facilitate healthcare services. The development of mHealth interventions (i.e., mobile devices to support medical and public health practice) is rapidly increasing, because it has the potential to be efficient, is accessible, safe, cost-effective, and adjustable to one’s preferences [4, 6, 7]. Moreover, 70% of the total population in Western Europe owns a smartphone [8], indicating that mHealth can target many patients with chronic conditions.

MHealth interventions seem to be in particular promising for specific patient groups such as adolescents, as adherence rates decrease during adolescence and almost all adolescents (95%) own a smartphone [9, 10]. During adolescence, patients start to develop their own medication beliefs and medication intake habits [11], which may persist into adulthood. It is therefore an important life phase for interventions aiming at medication use. However, most mHealth interventions are not intended for adolescents or targeted just one aspect of disease management [5, 12–15], e.g., a reminder to prevent forgetting, while previous studies showed that solely one element does not give sufficient support to children and adolescents [16]. We developed, in co-creation with adolescents with asthma [17], an interactive mHealth intervention with different components to support self-management; the ADolescent Adherence Patient Tool (ADAPT) [18]. The ADAPT intervention supported self-management, i.e., increased medication adherence of adolescents with asthma having poor adherence rates [19].

Further implementation and integration of mHealth in clinical practice is a complex process. Besides mHealth characteristics, the context plays an important role, such as the setting in which mHealth is used, the process of using mHealth, and the characteristics of the users [20]. Process evaluations and user experiences are therefore needed to increase the understanding of the implementation and integration of mHealth in clinical practice [21].

### Aim of the study

The aim of this study was to explore experiences, barriers, and facilitators of pharmacists and patients towards the ADAPT intervention, and to explore the perceptions of pharmacists towards mHealth interventions in general.

### Ethics approval

The current study is part of the ADAPT trial, which is approved by the Medical Review Ethics Committee of the University Medical Centre Utrecht (NL50997.041.14) and by the Institutional Review Board of Utrecht Pharmacy Practice network for Education and Research (UPPER), Department of Pharmaceutical Sciences, Utrecht University [22]. The trial is registered at the Dutch Trial Register (NTR5061). Before start of the study, all patients signed informed consent and for patients younger than 16 years, both parents also had to sign [18, 19].

## Method

### Study setting and participants

All pharmacists and patients participated in the ADAPT study; a 6-months cluster randomized controlled trial to test the effectiveness of the ADAPT intervention. The complete rationale, design, and effectiveness of the ADAPT study are described elsewhere [18, 19].

Patients (N = 87) who used the ADAPT intervention were invited to complete an online questionnaire to evaluate the ADAPT intervention. Community pharmacists who had access to the ADAPT intervention (N = 24) were interviewed with a structured questionnaire in order to obtain extensive information about the ADAPT intervention. In addition, pharmacists who did not have access to the ADAPT intervention (N = 26) were asked to complete an online questionnaire on their perceptions towards mHealth in the pharmacy. Data was collected between May 2016 and July 2017.

### ADAPT intervention

The ADAPT intervention was developed together with adolescents with asthma, and was based on the Common Sense Model of Self-Regulation [23]. The intervention consisted of an app for patients, which was connected to a desktop management system in the pharmacy. The ADAPT intervention was interactive and contained motivational, educational, and behavioural components (Table 1) to support self-management and adherence [18]. Patients were asked to complete the questionnaire to monitor symptoms at least once a week. Pharmacists received e-mail notifications when a patient possibly required care and they were asked to support the patient (when needed) by using the pharmacist chat.

**Table 1:** Components of the ADolescent Adherence Patient Tool (ADAPT), an interactive mHealth intervention consisting of a smartphone application (app) for patients connected to a desktop management system for pharmacists

Intervention component	Aim	Explanation
Weekly CARAT questionnaire	To monitor symptoms (motivational and educational)	Patients received a weekly reminder to complete this 10-item questionnaire on the app, which enables them (and their pharmacist) to monitor asthma and allergic rhinitis symptom over time
Medication reminder	To prevent forgetting (behavioural)	Patients could set an alarm once or twice a day, based on their medication regimen and their preferences
Movies	To educate and motivate	Patients received weekly movies on the app, additionally pharmacist could send specific movies to the patients app, e.g., concerning inhaler instructions
Peer chat	To facilitate contact	Patients could chat with peers; other asthma patients who participated in the study. This is an age-specific element, based on the adolescents’ preferences
Pharmacist chat	To facilitate contact (motivational and educational)	Patients and their pharmacists could send chat messages, e.g., for questions and feedback

*app* smartphone application, *CARAT* Control of Allergic Rhinitis and Asthma Test, *ADAPT* ADolescent Adherence Patient Tool

### Questionnaire for patients who had access to the intervention

The online questionnaire for patients was designed to evaluate patients’ experiences with the ADAPT intervention. The questionnaire contained open-ended and 5-point Likert scale questions (totally disagree to totally agree) on the use (ease and frequency), experiences with the different components (usefulness and enjoyability), and facilitators and barriers for using the intervention in everyday life [24]. Age, gender, self-reported medication use, adherence, and disease control of patients was registered. Personal data was encrypted using a study code, ensuring privacy of all participants.

### Questionnaire for pharmacists who had access to the intervention

Pharmacists were interviewed with a structured questionnaire by a research assistant, because the aim was to obtain extensive information on the ADAPT intervention. The structured questionnaire contained questions on pharmacy characteristics and on their experiences with the ADAPT intervention, i.e., about the use (ease and frequency), their experience with the different components, barriers and facilitators for use, and their perceptions on implementation and integration of the ADAPT intervention in clinical practice [24]. Additionally, pharmacists were asked to complete a short evaluation questionnaire using a 5-point Likert scale (totally disagree to totally agree).

### Questionnaire for pharmacists who *did not* have access to the intervention

Pharmacists who did not have access to the ADAPT intervention completed an online questionnaire on their perceptions towards mHealth in the pharmacy. This questionnaire contained open-ended, closed-ended, and 5-point Likert scale questions (not important to extremely important) on their previous experiences with mHealth, perceptions on different components, feasibility of mHealth, and barriers and facilitators for using mHealth in the pharmacy. These questions were not related to the ADAPT intervention. Moreover, these pharmacists provided basic pharmacy characteristics.

### Data analysis

Descriptive statistics were calculated, such as percentages and means with standard deviations (SD). Statistical analysis were performed using IBM SPSS Statistics for Windows, version 24.0.

## Results

### Patients about the ADAPT intervention

Of all patients who had access to the ADAPT intervention (N = 87), five patients reported no use of the intervention. The characteristics of the other 82 patients (users of the intervention) are shown in Table 2. Their mean age was 15.6 ± 2.0 years, 57.3% was female, and 59.8% (49/82) did not use the mHealth intervention for the complete 6-months study period. Main reasons for not using the intervention (at all) were forgetfulness (50.0%; 27/54) and technical issues (18.5%; 10/54).

**Table 2:** Characteristics of the patients who used the ADAPT intervention (N = 82)

	Patients % (n)
Female gender	57.3 (47)
Age, mean (SD)	15.6 (2.0)
Asthma medication use > 6 years	61.0 (50)
Adherent (MARS ≥ 23)	34.5 (30)
CARAT controlled (CARAT > 24)	22.0 (18)
Allergic rhinitis controlled (> 8)	36.6 (30)
Asthma controlled (≥ 16)	29.3 (24)

*ADAPT* ADolescent Adherence Patient Tool, *CARAT* Control of Allergic Rhinitis and Asthma Test, *MARS* Medication Adherence Report Scale, *SD* standard deviation

**Fig. 1:**
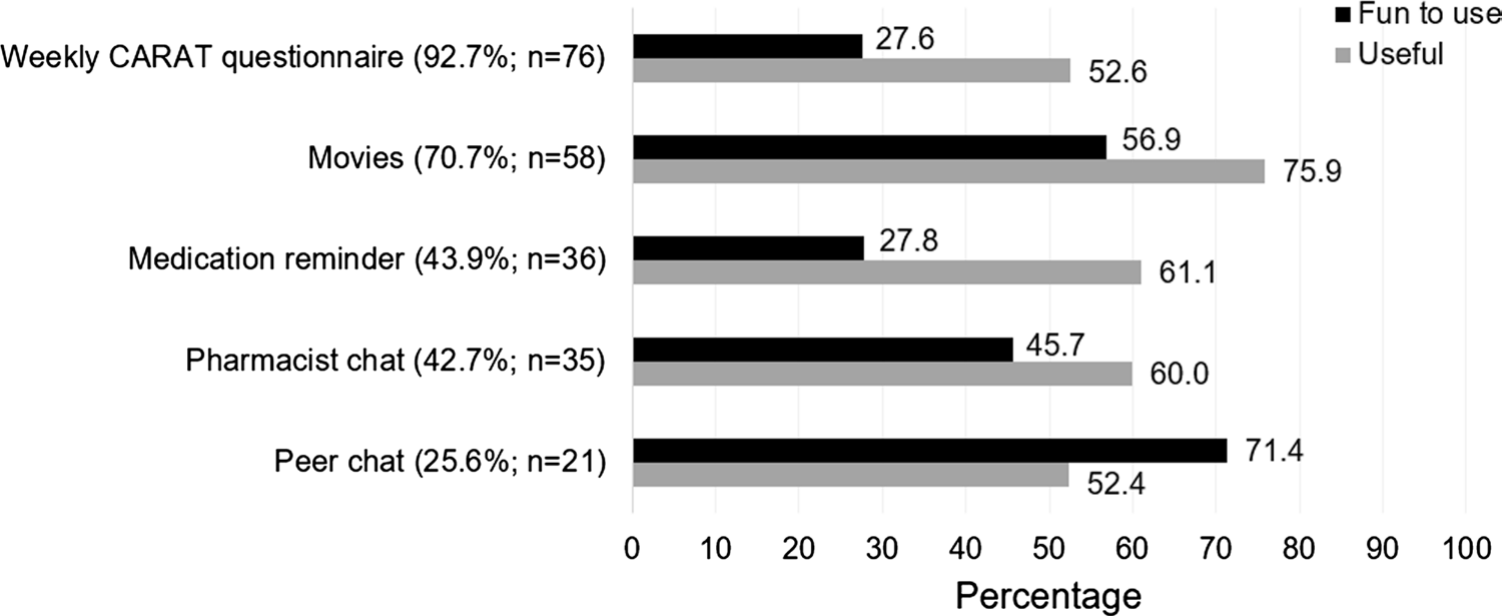
Self-reported use per component of the ADolescent Adherence Patient Tool (ADAPT), sorted from most to less used (N = 82), and the percentage of users who perceived the component as fun to use (black) or useful (grey). *CARAT* control of allergic rhinitis and asthma test

**Fig. 2:**
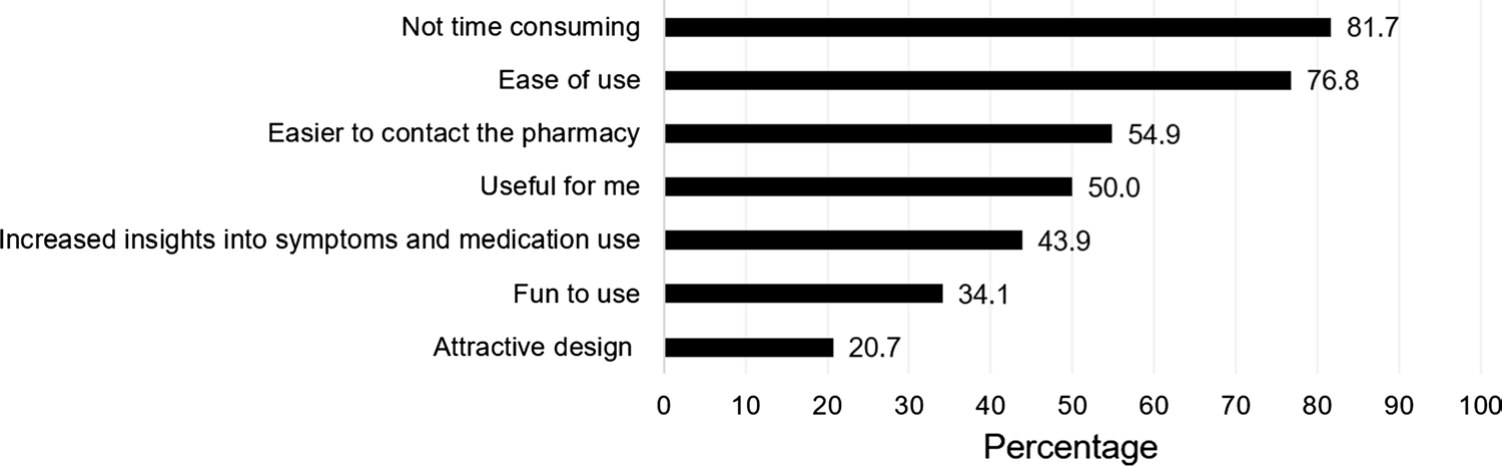
The percentage of patients (N = 82) who agreed (totally agree and agree) with the statements about the ADolescent Adherence Patient Tool (ADAPT)

The majority of patients (63.4%; 52/82) used the intervention at least once a week. The questionnaire to monitor symptoms (52.4%; 43/82) and the medication reminder (23.2%; 19/82) were appreciated most. The number of users and their opinion per intervention component is shown in Fig. 1. The weekly Control of Allergic Rhinitis and Asthma Test (CARAT) to monitor symptoms was used by most patients (92.7%; 76/82), thereafter the movies (70.7%; 58/82), which were regarded as useful by most users (75.9%; 44/58). The peer chat was observed as ‘fun to use’ by most users (71.4%; 15/21), however it was used by 25.6% (21/82) of the patients. Figure 2 shows the opinion of patients about the ADAPT intervention, suggesting that the intervention was not time consuming and easy to use.

The aim of the ADAPT intervention was to support self-management and increase adherence; 18.3% (15/82) of the patients reported to be more aware of their medication, and used their medication more regularly and more often. Problems with the mHealth intervention were experienced by 28.0% (23/82), which were mainly ICT related problems with the medication reminder or app crashes. Most patients (78.0%; 64/82) would recommend the ADAPT intervention to others, with ‘convenient’ as the main reason. In total, 63.4% (52/82) of the adolescents agreed that the pharmacy is the right place for providing treatment related information.

### Pharmacists who had access to the ADAPT intervention

Almost all pharmacists (95.8%; 23/24) used the ADAPT intervention, reasons for not using the intervention (n = 1) were not fitting with daily activities and preferably patient contact via mail (instead of an app). We excluded this pharmacist from further analyses. The characteristics of pharmacists who used the ADAPT intervention (N = 23) are shown in Table 3. Most pharmacists (73.9%; 17/23) used the mHealth intervention for the complete study period. Two participants were pharmacy technicians, who were specialised in pulmonary care. In three pharmacies more than one pharmacist was responsible for the intervention.

**Table 3:** Characteristics of the pharmacist study population

	Intervention group (N = 23) % (n)	Control group (N = 26) % (n)
**Pharmacist characteristics**		
Female gender	73.9 (17)	57.7 (15)
Age, mean (SD)	35.1 (9.0)	43.0 (8.8)
Working experience in years, mean (SD)	9.6 (8.1)	16.8 (8.4)
Previous experiences with mHealth	30.4 (7)	42.3 (11)
**Pharmacy characteristics**		
Number of pharmacists (FTE), mean (SD)	1.7 (0.6)	1.4 (0.6)
Number of pharmacy technicians (FTE), mean (SD)	6.4 (3.2)	6.1 (3.1)
Located in urban environment	65.2 (15)	65.4 (17)
Located in health center	65.2 (15)	73.1 (19)

*FTE* full time equivalent, *mHealth* mobile health, *SD* standard deviation

**Fig. 3:**
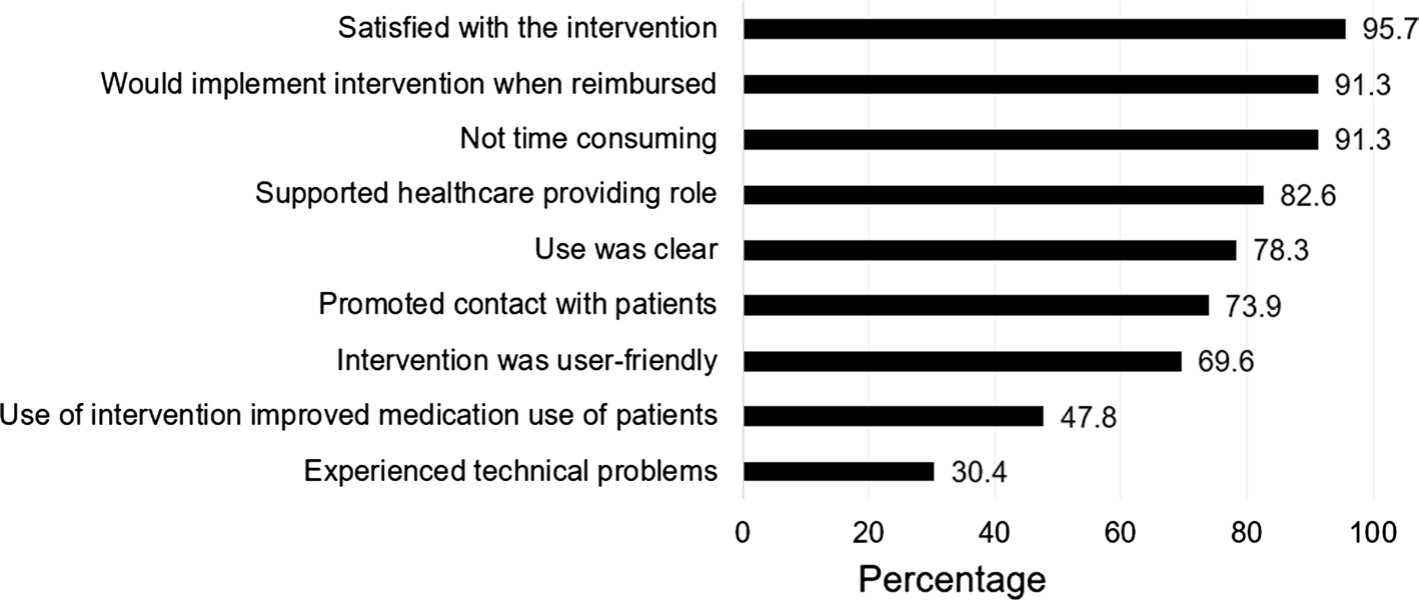
The percentage of pharmacists (N = 23) who agreed with the statements about the ADolescent Adherence Patient Tool (ADAPT)

Before the start of the intervention, more than half of the pharmacists (56.5%; 13/23) were not familiar with the use of electronic health (eHealth) in the pharmacy. During the ADAPT study, on average 3 ± 2 patients per pharmacy used the intervention. Using the intervention was not time consuming for most pharmacists (91.3%; 21/23; Fig. 3), varying from a few minutes to 20 min per week depending on the patient’s needs. The pharmacist with most participants (n = 8) spent on average 5 min per week on the intervention. Almost all pharmacists (95.7%; 22/23) were satisfied with the ADAPT intervention (Fig. 3), and 73.9% (17/23) contacted patients, based on e-mail notifications generated by the desktop management system, such as a low asthma control score or a question via the pharmacist chat.

The use of the intervention was clear for 78.3% (18/23) and the desktop management system was regarded as user-friendly by 69.6% (16/23) of the pharmacists (Fig. 3). The chat function with the patients and the questionnaire to monitor symptoms of the patient were appreciated most. For most pharmacists, the ADAPT intervention promoted contact with patients (73.9%; 17/23) and it supported the pharmacist’s role as a healthcare provider (82.6%; 19/23). In total, 47.8% of the pharmacists (11/23) thought that the intervention improved medication use of their patients (Fig. 3). However, the low number of patients per pharmacy, reluctance of patients, time constraints, and the non-intuitiveness of the intervention were reasons why the ADAPT intervention did not meet expectations for ten pharmacists (43.5%; 10/23). Moreover technical problems were experienced by 30.4% (7/23) pharmacists (Fig. 3), mainly related to updates of the desktop management system. Six pharmacists suggested an improvement in the usability of the intervention, e.g., easier login procedure. Integration of the desktop management system in the pharmacy information system would be a major improvement according to all pharmacists.

Most pharmacists (91.3%; 21/23) would implement the intervention when reimbursed (Fig. 3). However, there were concerns about the patient population, as adolescents were experienced as reluctant and hard-to-reach. Pharmacists suggested older patients with chronic diseases such as diabetes, asthma/COPD, or cardiovascular diseases as a target population. The majority of pharmacists (95.7%; 22/23) agreed that the pharmacy is the right place for mHealth interventions, like the ADAPT intervention, because medication counselling and adherence were seen as the responsibility of pharmacists. Moreover, the pharmacy was suggested as easy accessible. The reason for not using mHealth in the pharmacy was that patients might prefer their general practitioner as a healthcare provider, instead of their pharmacist.

### Pharmacists who *did not* have access to the ADAPT intervention

Characteristics of the 26 pharmacists who did not have access to the ADAPT intervention are shown in Table 3. More than half of the pharmacists (57.7%; 15/26) had never heard of mHealth interventions before. Two pharmacists used mHealth interventions previously, and almost all other pharmacists would like to use mHealth in their pharmacy (95.8%; 23/24).

Main expected facilitators for using mHealth were supporting adherence (84.6%; 22/26) and providing extra care for patients (80.8%; 21/26), while the main barriers were time constraints (53.8%; 14/26) and lack of reimbursement (46.2%; 12/26). Most pharmacists (80.8%; 21/26) thought they had sufficient skills to use mHealth, while a lack of mHealth knowledge was mentioned by others (n = 5). The majority of pharmacists (88.5%; 23/26) thought that innovations, such as mHealth, are needed to be prepared for the future.

The pharmacy was seen as the right place for mHealth interventions supporting medication use (92.3%; 24/26), because mHealth can support the healthcare providing role of pharmacists (87.5%; 21/24), medication counselling is seen as the responsibility of pharmacists (83.3%; 20/24), and the pharmacy might be more accessible than the general practitioner (66.7%; 16/24). Moreover, almost all pharmacists thought that mHealth could also be useful for other chronic diseases, such as diabetes (96.2%; 25/26) and cardiovascular diseases (92.3%; 24/26). Half of the pharmacists (50.0%; 13/26) thought that mHealth is also useful for non-chronic diseases to provide extra information and to ensure correct medication use, for example with antibiotics.

Funding was seen as an important factor for implementing mHealth in daily practice, because mHealth improves medication counselling (88.5%; 23/26), using mHealth costs time (73.1%; 19/26), and (electronic) consults should be reimbursed (50.0%; 13/26). All pharmacists (N = 26) would implement mHealth when reimbursed.

## Discussion

Pharmacists and patients were generally positive about the ADAPT intervention. Almost all pharmacists were satisfied with the intervention and the majority of patients would recommend it to others. Providing extra care for patients was one of the main reasons for using mHealth (by both pharmacist groups). Pharmacists who delivered the ADAPT intervention valued the improved patient contact. Negative experiences with the ADAPT intervention were mainly related to technical problems, due to updates, which might hamper further implementation of mHealth. However, updates are important to ensure the safety and privacy of mHealth. Technical issues should therefore receive high priority when further implementing mHealth. Another important facilitator for further implementation is the integration of mHealth in the pharmacy information system, because a ‘stand-alone’ desktop program restrained the integration with the pharmacist’s workflow. Although, the majority of pharmacists experienced the desktop management system as user-friendly and clear, which are important factors for acceptance and uptake [25].

The weekly questionnaire to monitor symptoms was the most frequently used mHealth component, and it was highly appreciated by patients and pharmacists. We used the CARAT questionnaire [26], which is a validated questionnaire consisting of ten questions on allergic rhinitis and asthma symptoms. Monitoring symptoms contributes to improved health outcomes [27] and based on the current positive perceptions, we recommend a short questionnaire as a useful component for mHealth interventions. Pharmacists also highly appreciated the possibility to chat with patients, while they experienced some non-response of patients. Chatting with patients, i.e., an electronic consultation (e-consult), provide patients with the opportunity to ask questions, while pharmacists can answer them when it fit with their daily activities. A unique aspect for patients is that they can re-read the consult when needed [28]. E-consults are new for patients and pharmacists, therefore more research should be conducted towards effective ways of digital communication with patients.

For both patients and pharmacists, the use of the ADAPT intervention was not time consuming, however time constraints were named as an important barrier for using mHealth by pharmacists who did not have access to the ADAPT intervention. For further implementation, it is therefore important to emphasize that the ADAPT intervention was not time consuming for 91.3% of the pharmacists. Moreover, integration of the desktop management system in the pharmacy information system will support efficient use of the intervention. Regardless of the efficient use, the ADAPT intervention might become more time consuming, when implemented among all adolescents with asthma. Because on average 18 adolescents per pharmacy use asthma medication [19], while in the ADAPT study on average three patients per pharmacy participated. Nonetheless, the time spend on the ADAPT intervention depended on the patient’s needs and the intervention should not be seen as something extra, instead it can replace other tasks, such as consultations and medication reviews, and can thereby potentially save time on the long-term.

In the current study, the pharmacy was seen as the right place for mHealth interventions, like the ADAPT intervention. In the Netherlands, every patient is registered at one pharmacy and mostly fill all their prescriptions there. As a medication expert and healthcare provider, pharmacists are responsible for medication counselling and adherence. They can thereby improve the quality of patient care and outcomes. MHealth interventions can facilitate the pharmacist’s responsibilities and promote contact with patients. This is important nowadays, because pharmacists are expected to combine their management role with more healthcare providing roles, and there is an ongoing shift towards integrated care settings [29]. Currently, not many mHealth interventions are designed in pharmacies [4, 6, 30], while positive effects of pharmacy delivered mHealth interventions are shown for disease management of several chronic diseases in adult patients [31, 32]. In the current study, even non-chronic medication users were mentioned as a target group for mHealth. Therefore further research should focus on the implementation and integration of mHealth in pharmacy practice [31].

Intuitive usability and clear explanations of mHealth intervention were suggested to support usability and are therefore important for further implementation. A previous study also showed the importance of training for using mHealth interventions [25]. However, firstly, pharmacists should be aware of the possibilities for mHealth in the pharmacy, because in the current study only a minority of pharmacists were familiar with mHealth and/or eHealth. Moreover, pharmacy students would like to recommend mHealth to their future patients [33], i.e., there is room for improvement.

All pharmacists and patients voluntary participated in the ADAPT study and might therefore be more enthusiastic and positive about mHealth, or more motivated to use the ADAPT intervention. Thus, the current study might contain a response bias. Nonetheless, this evaluation study provides insights into the perceptions of patients and pharmacists about a mHealth intervention, and it highlighted main barriers and facilitators for using mHealth in a pharmacy setting. This is important for (research towards) further implementation and integration of mHealth in clinical practice. Our exploratory findings should be taken into account when developing mHealth interventions to support self-management and adherence. However, more research is needed towards the evaluation of mHealth interventions in the pharmacy to generalize our findings and towards the cost-effectiveness of mHealth, which is important for the development of reimbursement guidelines.

## Conclusion

Both patients and pharmacists perceived beneficial effects and were positive about the ADAPT intervention. The intervention was not time consuming, while time constraints were expected barriers by pharmacists who did not deliver the ADAPT intervention. Moreover, the ADAPT intervention facilitated the pharmacist’s role as a healthcare provider and promoted contact with patients. Attention should be paid to prevent technical issues and to ensure reimbursement guidelines. The pharmacy setting was seen as a right place for mHealth interventions supporting appropriate medication use, also for patients other than asthma patients. This study emphasizes the opportunities for mHealth in improving the quality of care, and the current findings should be emphasized among pharmacists, other healthcare providers, and intervention developers. Further research should focus on generalizability of our findings and on the further implementation and integration of mHealth in the (pharmacy) healthcare setting.

## Electronic supplementary material

Below is the link to the electronic supplementary material.

Supplementary material 1 (DOCX 30 kb)
