# Supplementary material for: Evaluation of a mobile health intervention to support asthma self-management and adherence in the pharmacy
Source: Int J Clin Pharm. 2019 Apr 27;41(2):452–9. doi: 10.1007/s11096-019-00798-3 (PMC6509217; doi:10.1007/s11096-019-00798-3)
Supplement: Supplementary file 1 — Supplementary material 1 (DOCX 30 kb) [file 11096_2019_798_MOESM1_ESM.docx]

**Appendix 1.** **The questionnaires used to evaluate the ADAPT intervention**

**1. Questionnaire for patients who had access to the intervention**

1. **Experiences with the ADAPT intervention**
2. How long did you use the ADAPT intervention during the study period? Please explain.
3. How often did you use the ADAPT intervention?
4. On average, how many minutes per week did you spend on using the ADAPT intervention?
5. Did the ADAPT intervention meet your expectations? Please explain.
6. **The ADAPT application**
7. Did you use the weekly questionnaire to monitor your symptoms?

If no: please explain.

If yes:

- It was useful to complete the weekly questionnaire.

*5-point Likert scale (totally disagree to totally agree)*

- It was fun to complete the weekly questionnaire.

*5-point Likert scale (totally disagree to totally agree)*

1. Did you use the medication reminder?

If no: please explain.

If yes:

- It was useful to use the medication reminder.

*5-point Likert scale (totally disagree to totally agree)*

- It was fun to use the medication reminder.

*5-point Likert scale (totally disagree to totally agree)*

1. Did you use the peer chat?

If no: please explain.

If yes:

- It was useful to use the peer chat.

*5-point Likert scale (totally disagree to totally agree)*

- It was fun to use the peer chat.

*5-point Likert scale (totally disagree to totally agree)*

1. Did you use the pharmacist chat?

If no: please explain.

If yes:

- It was useful to use the pharmacist chat.

*5-point Likert scale (totally disagree to totally agree)*

- It was fun to use the pharmacist chat.

*5-point Likert scale (totally disagree to totally agree)*

1. Did you watch the movies?

If no: please explain.

If yes:

- It was useful to watch the movies.

*5-point Likert scale (totally disagree to totally agree)*

- It was fun to watch the movies.

*5-point Likert scale (totally disagree to totally agree)*

1. Do you have suggestions for movie topics? If yes, please explain.
2. Which component of the intervention was most useful?

Weekly CARAT questionnaire; medication reminder; peer chat; pharmacist chat; movies.

*Please place them in order from 1 (most useful) to 5 (least useful).*

1. **The ADAPT intervention**
2. Did you miss components in the ADAPT application? Please explain.
3. Did you adjust your medication intake behaviour due to the app? Please explain.
4. How often did you receive information from your pharmacist in the app?
5. Would you recommend the ADAPT intervention to others? Please explain.
6. Did you experience any problems or issues while using the intervention? Please explain.
7. Do you have other comments/suggestions? Please explain.
8. **Statements about the use of the ADAPT intervention**

| I like to have contact with the pharmacy through the app. | Totally disagree | Disagree | Neutral | Agree | Totally agree |
| --- | --- | --- | --- | --- | --- |
| Using the app was useful to me. |  |  |  |  |  |
| Using the app was fun. |  |  |  |  |  |
| The pharmacist knows too little about my personal situation to support me. |  |  |  |  |  |
| I would have preferred another way of receiving information from the pharmacy. |  |  |  |  |  |
| Using the app made it easier to contact the pharmacy. |  |  |  |  |  |
| I gained more insights into my respiratory symptoms and my mediation use. |  |  |  |  |  |
| I changed my medication use, due to the app. |  |  |  |  |  |
| The app has an attractive design. |  |  |  |  |  |
| It was easy to use the app. |  |  |  |  |  |
| Using the app was not time consuming. |  |  |  |  |  |
| I used the app and the information in the app a lot. |  |  |  |  |  |
| The pharmacy is the right place to provide information to adolescents with asthma. |  |  |  |  |  |

**2. Questionnaire for pharmacists who had access to the intervention (structured interview)**

1. **Experiences with the ADAPT intervention in the pharmacy**
2. How long did you use the ADAPT intervention during the study period? Please explain.
3. Did the ADAPT intervention meet your expectations? Please explain.
4. Did you miss components in the ADAPT intervention? Please explain.
5. Which component of the ADAPT intervention was the most useful? Please explain.
6. Which component of the intervention was most useful? Please explain.

CARAT table; CARAT graph; intervention guide; status overview; e-consult; movies

*Please place them in order from 1 (most useful) to 7 (least useful).*

1. How many patients did you guide during the ADAPT study?
2. How many minutes per week did you spend on using the ADAPT intervention?
3. How many minutes per week did you spend on the ADAPT intervention per patient?
4. How often did you send information to patients, or did you contact them? Please explain.
5. Did you experience technical problems or issues? Please explain.
6. Did you experience other problems? Please explain.
7. Would you like to use the intervention in clinical practice when reimbursed?

If no: please explain.

If yes: for which patients?

1. What is a reasonable price for the mHealth guidance of a patient for one year?
2. What do you think about an integration of the mHealth intervention in the pharmacy information system? Please explain.
3. Do you think the pharmacy is the right place for mHealth interventions like ADAPT? Please explain.
4. Do you have comments/suggestions for the implementation of ADAPT in clinical practice? Please explain.
5. Were you satisfied with ADAPT? Please explain.
6. **Pharmacy characteristics**
7. On average, how many pharmacists work in your pharmacy per day?
8. On average, how many pharmacy technicians work in your pharmacy per day?
9. Is your pharmacy located in a health centre?
10. Do you participate in pharmacotherapy consultations (FTO)?
11. In which province is your pharmacy located?
12. Is your pharmacy located in an urban or rural area?
13. **Pharmacist characteristics**
14. Did other colleagues also used the ADAPT intervention? Please explain.
15. What is your position within the pharmacy?
16. How many working experience do you have (in years)?
17. What is your age?
18. Are you male or female?
19. Do you have other comments/suggestions regarding the research project?
20. **Statements about the use of the ADAPT intervention**

| Before the start of the study I was familiar with using eHealth interventions in the pharmacy. | Totally disagree | Disagree | Neutral | Agree | Totally agree |
| --- | --- | --- | --- | --- | --- |
| Adolescents are difficult to reach. |  |  |  |  |  |
| Use of the ADAPT intervention results in improved medication use of patients. |  |  |  |  |  |
| I gained more insights into respiratory complaints and medication use of the patients who participated. |  |  |  |  |  |
| Use of the ADAPT intervention promoted communication between adolescents and the pharmacy. |  |  |  |  |  |
| Use of the ADAPT intervention was clear for the patient. |  |  |  |  |  |
| Use of the ADAPT intervention was clear to me. |  |  |  |  |  |
| During the study, I contacted patients based on information received of the intervention. |  |  |  |  |  |
| The desktop management system was user friendly. |  |  |  |  |  |
| I required extra training to implement the intervention. |  |  |  |  |  |
| The training on asthma in adolescents was useful. |  |  |  |  |  |
| The guidance of patients through the intervention was not time consuming. |  |  |  |  |  |
| The ADAPT intervention supported the pharmacy in guiding patients with their medication use. |  |  |  |  |  |

**3. Questionnaire for pharmacists who *did not* have access to the intervention**

1. **mHealth interventions in general**
2. Would you like to use a mHealth intervention in your pharmacy?

If no: please explain

If yes: What components should a mHealth intervention contain? *(multiple answers possible)*

- Chat function with the patient

- Chat function with the physician

- Diary to monitor symptoms

- Medication reminder/alarm

- Questionnaire about the patient’s health status

- Instruction movies

- Reminder for medication prescription

- Other:….

1. Whether you do, or do not, want to use mHealth in the pharmacy: what are main reasons to use mHealth? *(multiple answers possible)*

- Supporting adherence of patients
- Reaching patients who don’t visit the pharmacy
- Create more awareness among the patients about the disease

- Create more awareness among the patients on the need for medication use

- The possibility to provide more care

- Financial reasons

- Other: ….

1. Whether you do, or do not, want to use mHealth in the pharmacy: what are main reasons *not to*
   use mHealth? *(multiple answers possible)*

- Financial reasons
- No added value of mHealth

- Time consuming

- Patients are not ready for it

- Patients receive enough support in the pharmacy

- Insufficient knowledge

- Other: ….

1. Have you ever worked with mHealth intervenions? Please explain.
2. Is the pharmacy the right place for a mHealth intervention like ADAPT? Please explain.
3. Are questionnaires a suitable way to obtain information about the health status of patients? Please explain.
4. Innovations, like mHealth, are necessary to be prepared for the future.

*5-point Likert scale (totally disagree to totally agree)*

1. How important are the following aspects of a mHealth intervention for you?

| Usability. | Not important | Not very important | Fairly important | Very important | Extremely important |
| --- | --- | --- | --- | --- | --- |
| Informing the patient about their condition and/or medication use. |  |  |  |  |  |
| Quickly contacting the patient, for example by using a chat function. |  |  |  |  |  |
| Use of photos or movies to inform patients. |  |  |  |  |  |
| Supporting patients with their medication use. |  |  |  |  |  |
| Following the patient over time, for example by using a symptom monitor. |  |  |  |  |  |
| Integration of mHealth in the pharmacy information system. |  |  |  |  |  |
| The time it takes to use the intervention. |  |  |  |  |  |
| The reimbursement guidelines. |  |  |  |  |  |
| The costs of mHealth. |  |  |  |  |  |

1. **Implementation of mHealth**
2. Would you like to use a mHealth intervention when reimbursed? Please explain.
3. Do you have sufficient skills to use mHealth? Please explain.
4. What is a reasonable amount of time to spend per week on the guidance of a patient? Please explain.
5. What is a reasonable reimbursement price for the guidance of a patient for one year?
6. Is mHealth suitable for patients with different ages? Please explain.
7. Is mHealth suitable for patients with other chronic conditions? Please explain.
8. Is mHealth suitble for patients with non-chronic conditions? Please explain.
9. **General characteristics**
10. What is your position within the pharmacy?
11. What is your age?
12. Are you male or female?
13. How many working experience do you have (in years)?
14. On average, how many pharmacists work in your pharmacy per day?
15. On average, how many pharmacy technicians work in your pharmacy per day?
16. Is your pharmacy located in a health centre?
17. Do you participate in pharmacotherapy consultations (FTO)?
18. In which province is your pharmacy located?
19. Is your pharmacy located in an urban or rural area?
